# Tracing epistemic injustice in global antimicrobial resistance research

**DOI:** 10.1016/j.tim.2025.02.005

**Published:** 2025-06

**Authors:** Phaik Yeong Cheah, Sonia Lewycka, Jantina de Vries

**Affiliations:** 1 Mahidol Oxford Tropical Medicine Research Unit, Faculty of Tropical Medicine, Bangkok, Thailand; 2 Nuffield Department of Medicine, University of Oxford, Oxford, UK; 3 Oxford University Clinical Research Unit, Hanoi, Viet Nam; 4 The Ethics Lab, University of Cape Town, Cape Town, South Africa

**Keywords:** epistemic injustice, antimicrobial resistance, ethics, equity, diversity, inclusion

## Abstract

This commentary explores whether there is epistemic injustice in global antimicrobial resistance (AMR) research – who sets priorities, who produces knowledge, and which types of knowledge are valued. We argue that epistemic injustice may have created blind spots in policy. Addressing this requires a commitment to diversity, equity, and inclusion.

## Introduction

AMR is a major global health threat that disproportionately impacts disadvantaged populations, particularly the poorest and most marginalized [1]. Despite this, the voices, values, data, and needs of these communities are often either under-recognised or excluded from AMR research.

This commentary focuses on one particular form of injustice in AMR research, namely epistemic injustice. Other forms of injustice – such as procedural and distributive injustice – have been more widely discussed in the literature and are not addressed here. In this paper, AMR research refers to research on prevention, diagnosis, and treatment of antimicrobial-resistant infections, as well as AMR epidemiology, burden, driver, and policies primarily funded by international funders. Although important, our commentary does not discuss AMR research conducted by pharmaceutical companies because such research is usually driven by commercial goals.

Epistemic injustice is injustice relating to the production and use of knowledge [2], and it ‘draws attention to inequalities in the knowledge that is valued and produced in today’s world, including emphases on technical and quantitative measures over qualitative measures rooted in lived experiences, and ‘expert’ scientific knowledge over local and indigenous ways of knowing’ [3]. Such injustices can take place along the lines of gender, geographic location, language, race and ethnicity, but also types of knowledge and ways of knowing, including those of indigenous populations. There is increasing focus on the process of justice (or injustice) that surrounds knowledge, that is, what we know, how we know it, who that knowledge is useful for, and whose interests it serves. Epistemic injustice has been described broadly for the field of global health [4], including global health ethics. Pratt and de Vries proposed that epistemic justice affects three layers of knowledge in global health ethics: at the level of knowledge produced; knowledge solicited; and knowledge applied. Drawing on that work, we propose that AMR research is troubled by similar instances of epistemic justice [3].

## Epistemic injustice in global AMR research

Here, we highlight three overlapping domains in which we consider epistemic injustice to occur in AMR research.

(i) Who sets global AMR research priorities? One important way in which epistemic justice seems to occur in AMR research, relates to how global AMR research priorities are set. Global health research priorities are set largely by both the World Health Organisation (WHO) and international funding bodies that support AMR research globally. Those priority-setting exercises do not normally include substantive representation by people from lower- and middle-income countries (LMICs) – whether they be researchers, clinicians, policy makers, or people from affected communities. In a recent WHO AMR research priority-setting committee, the authors acknowledged that ‘individuals from the WHO European region and region of the Americas were over-represented compared with those from other regions, reflecting a stronger investment in academic research in these two WHO regions’ [5]. Selection for such committees, as well as for AMR research funding review committees, is frequently influenced by the relative academic standing and prominence or visibility of researchers. The reasons may be tied to fluency in English which impacts the language of publication. Researchers who are less fluent in English, or who publish in languages other than English, may not be as internationally recognized or networked. The ‘grey literature’ that they produce, such as National Action Plans for AMR, may not be considered when looking for committee members or experts. When AMR research priority-setting committees do not include a representative balance of people, there is a risk that the kind of research that is pursued is the research that people in high-resource settings – who experience a different kind of AMR burden – recognize as important. Research that responds to, or considers, the particular way in which AMR manifests in different contexts in low-resource settings may not be recognized as important, or funded. This is evident from investments in AMR research – most funding focuses on basic research and the development of new antibiotics which may be less relevant to the research priorities of LMICs [6].

(ii) Who produces, interprets, and uses AMR knowledge? While the impact of AMR disproportionately affects people in LMICs [1], AMR research tends to be dominated by academics from higher-income countries (HICs) and by a small number of disciplinesi. There is also likely a gender imbalance: research has shown that women are under-represented as authors in American Society of Microbiology journals [7]. It is therefore likely that women are also under-represented in AMR research and in the production of AMR knowledge.

Furthermore, as in other fields, researchers from low-resource settings face significant barriers to publishing in high-impact journals. Language challenges can hinder their ability to produce competitive manuscripts, and limited access to protected, paid research time further constrains their capacity to write. Many LMIC clinician-researchers spend the majority of their time in clinical practice, leaving little opportunity for research and academic writing.

The resulting imbalance in publication numbers perpetuates epistemic injustice in two ways: on the one hand it limits opportunities for researchers from LMICs to shape global AMR knowledge; on the other hand, it means that the experiences, knowledge, and opportunities for AMR interventions that could originate from low-resource settings are not used in that way.

(iii) What knowledge is currently available and valued? A major gap in AMR research is the lack of data from LMICs – particularly in medically underserved areas and communities [1]. This can result in surveillance strategies that are non-representative of the target population [8]. Significant data gaps or biases in the prevalence and burden of AMR hinder reliable estimates, particularly in regions with inadequate laboratory capacity and data collection systems, and this might lead to inappropriate responses.

There is a significant lack of data about health-seeking behaviours, health practices, and medication use from many communities, including the most marginalised or vulnerable populations around the world. This gap exists partly because such data may be difficult to obtain; instead, the focus may be on populations that are more accessible or better represented in formal healthcare systems. Additionally, much of the knowledge and practices within these communities may be rooted in oral traditions or embedded in local cultural contexts, making it less likely to be documented in a form that aligns with the conventions of academic research. Even when such data are collected, they are often not readily available or may not be presented or discussed in English-language academic publications, which means that the knowledge is neither recognised nor incorporated in global discourse around AMR.

## Consequences of epistemic injustice in global AMR research

The epistemic injustices outlined in the preceding text contribute to what some scholars describe as ‘blind spots’ in AMR research and policy [9]. This is likely a consequence of existing imbalances of power among global health researchers and institutions, and the framing of global health threats as security concerns [10], which focuses on containment, surveillance, and preparedness. These blind spots include an excessive focus on HICs, human antibiotic overuse, hospital-based antibiotic stewardship, and antimicrobial drug development [9]. Relatively little attention is given to critical LMIC issues, such as reducing infection burden, antibiotic overuse in livestock farming, prescribing practices among primary care providers, and developing affordable, rapid diagnostic tests for detecting AMR [6,9].

Epistemic injustice could also mean that well-meaning interventions to curb AMR could have unintended consequences for some communities [11]. For example, the introduction of malaria rapid diagnostic tests, aimed at reducing unnecessary antimalarial prescriptions, has inadvertently led to increased antibiotic prescribing in Afghanistan, Uganda, and Tanzania [12]. Our own experience has shown that AMR awareness campaigns have sometimes instilled fear around the use of antibiotics and unfairly stigmatized small-scale farmers.

## Suggestions for the way forward

We suggest ways to address epistemic injustice in global AMR research. First, it is essential to understand the problem of representation in terms of global AMR research agenda setting. Shaping the research agenda should involve diverse researchers and practitioners to ensure that global research priorities align with the needs of affected communities. Specifically, what is required is ensuring that the composition of committees involved in priority-setting exercises is balanced in terms of geographical representation, high- versus low-resource settings, area of professional practice, disciplines, and gender.

Second, there is a need to ensure that diverse populations are included in research studies to generate data that accurately reflect the experiences of all groups, particularly those most affected by AMR. This should include communities normally under-served by research and under-included due to traditional notions of vulnerability. This includes removing barriers to research participation wherever possible and recognising the agency of individuals to participate in research.

Third, it is important to promote diversity, equity, and inclusion within research teams. Addressing AMR effectively requires solutions that extend beyond biomedical or technical interventions to include disciplines such as bioethics, law, human rights, and economics, ensuring a more holistic approach to the problem.

Fourth, community engagement around AMR research – such as using the ‘responsive dialogues’ approach – is vital to ensure that the voices of those most impacted by AMR are heard, helping to make research more responsive [13].

Fifth, equitable partnerships between HIC and LMIC institutions and researchers from various disciplines can help to balance power dynamics in AMR research. Funding agencies should support initiatives that encourage LMIC leadership.

Finally, we propose a ‘just transitions’ approach, adapting the concept that is increasingly used in climate-change mitigation in relation to policies aiming to shift toward more sustainable, low-carbon economies in a fair way [14]. A ‘just transition’ for AMR mitigation should ensure that solutions prioritise sustainability, inclusivity, equity, and justice, including epistemic justice [15].

## Funding

This research was funded in part by the Wellcome Trust (220211/Z/20/Z, 228141/Z/23/Z, 222870/Z/21/Z). For the purpose of open access, the authors have applied a CC BY public copyright licence to any author-accepted manuscript version arising from this submission. The funder had no role in the writing or preparation of the manuscript. P.Y.C. and S.L. are funded by the British Academy (GCPS2\100009) to participate in ‘A Just Transitions Framework for The Equitable and Sustainable Mitigation of Antimicrobial Resistance’ project.

## Declaration of interests

The authors declare no competing interests.

## Declaration of generative AI and AI-assisted technologies in the writing process

During the preparation of this work the authors used ChatGPT for proofreading. After using this tool/service, the authors reviewed and edited the content as needed and take full responsibility for the content of the publication.

## Resources

^i^ https://dzifhelmholtzdashboard.azurewebsites.net/reports/investments/overview
